# Complete Genome Sequence of *Stenotrophomonas* sp. Strain WZN-1, Which Is Capable of Degrading Polybrominated Diphenyl Ethers

**DOI:** 10.1128/genomeA.00722-17

**Published:** 2017-08-03

**Authors:** Zhineng Wu, Mark Bartlam, Yingying Wang

**Affiliations:** a Key Laboratory of Pollution Processes and Environmental Criteria (Ministry of Education), Tianjin Key Laboratory of Environmental Remediation and Pollution Control, College of Environmental Science and Engineering, Nankai University, Tianjin, China; b State Key Laboratory of Medicinal Chemical Biology, Nankai University, Tianjin, China; c College of Life Sciences, Nankai University, Tianjin, China

## Abstract

*Stenotrophomonas* sp. strain WZN-1, isolated from an e-waste recycling area in Tianjin, China, is capable of degrading polybrominated diphenyl ethers (PBDEs). The complete genome of strain WZN-1 consists of 4,512,703 bp. This genome information will provide important information about the biodegradation pathways and mechanisms of PBDEs.

## GENOME ANNOUNCEMENT

Polybrominated diphenyl ethers (PBDEs) are extensively used in various types of electronic equipment, furniture, and other products due to their excellent flame-retardant performance (1). PBDEs have become widespread environmental pollutants and are ubiquitous in the environment (2). They pose a great threat to both human health and the global ecosystem. Information on the microbial degradation of PBDEs is still limited. *Stenotrophomonas* sp. strain WZN-1 was isolated from soil samples at an e-waste recycling area located in Tianjin, China. It can utilize different PBDE congeners as its sole carbon source and degrade PBDEs efficiently under aerobic conditions.

The genomic DNA of strain WZN-1 was extracted using a PowerSoil DNA isolation kit (Mo Bio, USA). The complete genome sequencing of WZN-1 was carried out on the Illumina HiSeq 4000 and PacBio RSII sequencing platforms. All clean reads were assembled using SMRT Analysis version 2.3. The quality of the sequencing read data was estimated by G+C content and sequencing depth correlation analysis. The genes from the assembled result were predicted using Glimmer version 3.02 (3). For highly complex regions, PCRs were conducted to fill all the gaps. The genome size of strain WZN-1 is 4,512,703 bp with a G+C content of 66.62%. The genome contained 4,137 genes with a total length of 3,961,347 bp, which makes up 87.78% of the genome. The number of tandem repeat sequences was 253 with a total length of 19,245 bp, which makes up 0.4265% of the genome; the number of minisatellite DNAs was 167; the number of microsatellite DNAs was 29; and the numbers of tRNAs, rRNAs, and small RNAs (sRNAs) were 73, 13, and 1, respectively. The taxonomic position showed that strain WZN-1 was a member of the *Stenotrophomonas* subgroup in the class *Gammaproteobacteria*. Members of the genus *Stenotrophomonas* have been reported as promising candidates for biotechnological applications involved in the detoxification of various man-made pollutants (4), including polycyclic aromatic hydrocarbons (5), benzene, toluene, and ethylbenzene (6), heavy metals (7), and xenobiotics (8). *Stenotrophomonas* spp. have great application value in the field of environmental remediation and biodegradation of pollutants (9).

The open reading frames (ORFs) and the functional annotation of translated ORFs were predicted and achieved by subjecting the genes to a BLAST search in the following databases: Kyoto Encyclopedia of Genes and Genomes (KEGG), Cluster of Orthologous Groups of Proteins (COG), Swiss-Prot, NR, Gene Ontology (GO), Pathogen Host Interactions (PHI), Antibiotic Resistance Genes Database (ARDB), Virulence Factors of Pathogenic Bacteria (VFDB), and Carbohydrate-Active enZYmes Database (CAZy). Based on the functional annotation of the genes of strain WZN-1, the relevant genes involved in the degradation of PBDEs were located and analyzed. Genome annotation indicated that WZN-1 has a variety of metabolic pathways for aromatic compound degradation, xenobiotic biodegradation and metabolism, including haloacid dehalogenase and aromatic ring-opening dioxygenase, as well as some aromatic compound degradation metabolism.

In conclusion, we have sequenced the complete genome of *Stenotrophomonas* sp. strain WZN-1, which is capable of degrading PBDEs under aerobic conditions. This genome information will provide important information for understanding the molecular mechanisms of PBDE biodegradation.

### Accession number(s).

The complete genome sequence of *Stenotrophomonas* sp. strain WZN-1 has been deposited in GenBank under accession number CP021768.
